# Treatment of COVID-19 Pneumonia and Acute Respiratory Distress With Ramatroban, a Thromboxane A_2_ and Prostaglandin D_2_ Receptor Antagonist: A Four-Patient Case Series Report

**DOI:** 10.3389/fphar.2022.904020

**Published:** 2022-07-22

**Authors:** Martin L. Ogletree, Kate Chander Chiang, Rashmi Kulshrestha, Aditya Agarwal, Ashutosh Agarwal, Ajay Gupta

**Affiliations:** ^1^ Department of Pharmacology, Vanderbilt University School of Medicine, Nashville, TN, United States; ^2^ Charak Foundation, Orange, CA, United States; ^3^ Regulatory Wisdom, Delhi, India; ^4^ Charak Foundation of India, Indore, India; ^5^ EyeSight Eye Hospital and Retina Centre, Indore, India; ^6^ Division of Nephrology, Hypertension and Kidney Transplantation, University of California, Irvine, CA, United States

**Keywords:** SARS-CoV-2, COVID-19, ARDS, hypoxemia, thromboxane A2, ramatroban, pulmonary hypertension, pneumonia

## Abstract

Hypoxemia in COVID-19 pneumonia is associated with hospitalization, mechanical ventilation, and mortality. COVID-19 patients exhibit marked increases in fatty acid levels and inflammatory lipid mediators, predominantly arachidonic acid metabolites, notably thromboxane B_2_ >> prostaglandin E_2_ > prostaglandin D_2_. Thromboxane A_2_ increases pulmonary capillary pressure and microvascular permeability, leading to pulmonary edema, and causes bronchoconstriction contributing to ventilation/perfusion mismatch. Prostaglandin D_2_-stimulated IL-13 production is associated with respiratory failure, possibly due to hyaluronan accumulation in the lungs. Ramatroban is an orally bioavailable, dual thromboxane A_2_/TP and prostaglandin D_2_/DP2 receptor antagonist used in Japan for allergic rhinitis. Four consecutive outpatients with COVID-19 pneumonia treated with ramatroban exhibited rapid relief of dyspnea and hypoxemia within 12–36 h and complete resolution over 5 days, thereby avoiding hospitalization. Therefore, ramatroban as an antivasospastic, broncho-relaxant, antithrombotic, and immunomodulatory agent merits study in randomized clinical trials that might offer hope for a cost-effective pandemic treatment.

## 1 Introduction

After symptomatic SARS-CoV-2 infection, 10–20% of patients require hospitalization for respiratory distress and hypoxemia (Trust for American’s Health, 2021). Currently, anti-SARS-CoV-2 monoclonal antibodies are approved for treatment of ambulatory patients with COVID-19 (NIH, 2021), and antiviral treatments have recently been approved, but they are expensive and effective only early after symptom onset. There is an unmet medical need for inexpensive, safe, orally bioavailable drugs that can reduce hypoxemia, provide symptomatic relief, and minimize hospitalization in patients with COVID-19. Identifying the correct therapeutic target is critical to discovering such a drug.

**FIGURE 1:**
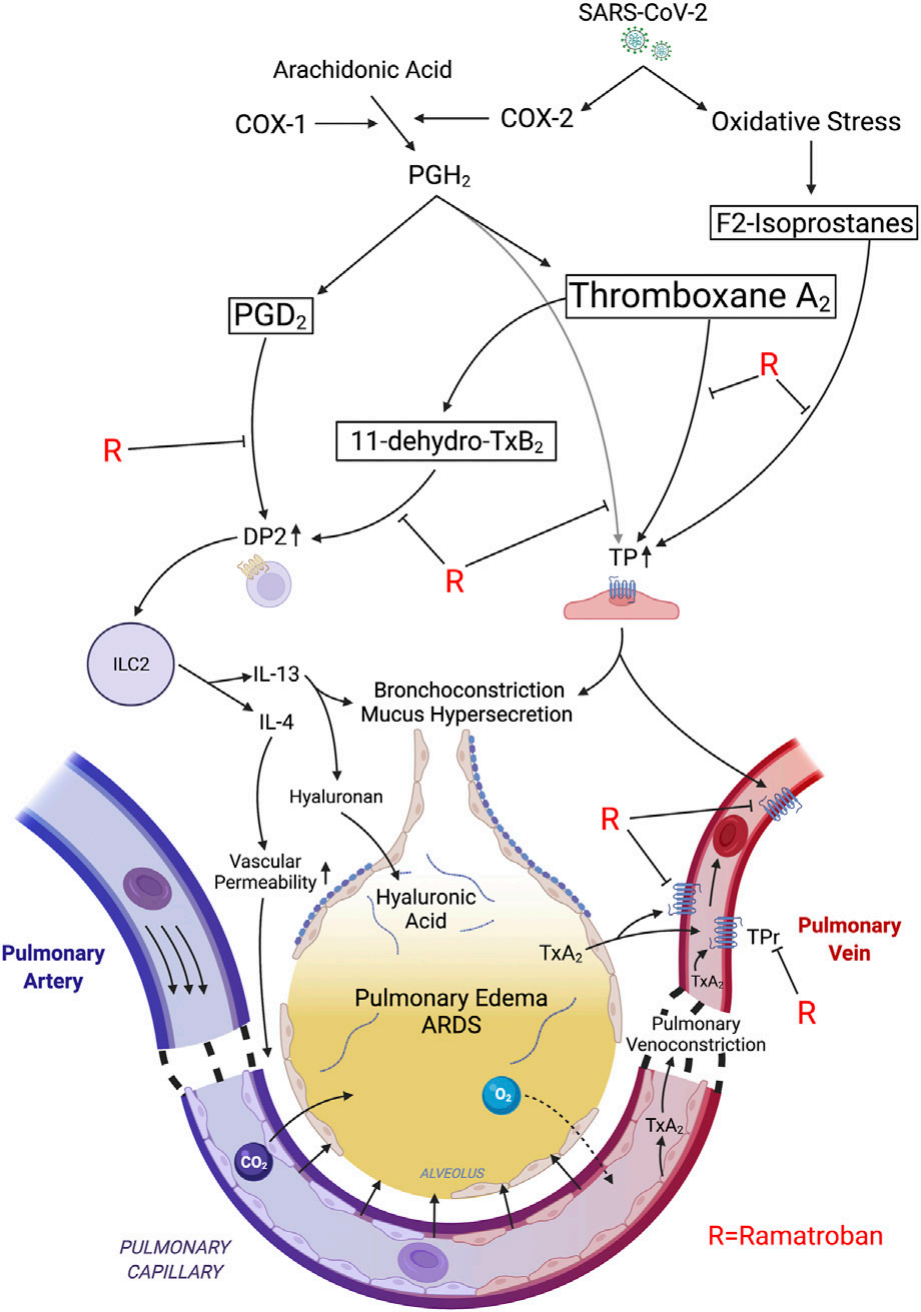
Proposed mechanisms of rapid relief in respiratory distress following ramatroban administration during acute SARS-CoV-2 infection. SARS-CoV-2 induced expression of COX-2 generates PGH_2_ which is converted into thromboxane A_2_ >> PGD_2_. Oxidative stress-associated free radicals initiate nonenzymatic peroxidation of arachidonic acid leading to F2-isoprostane generation. PGH_2_, TxA_2_, and F2-isoprostanes stimulate thromboxane prostanoid receptors (TP). TP stimulation induces pulmonary venoconstriction leading to an increase in transcapillary pressure in pulmonary microvasculature, and transudation of fluid into the alveoli, thereby causing impaired gas exchange and ARDS. The TxA_2_/TP axis also induces bronchoconstriction and mucus secretion. TxA_2_ is rapidly converted to 11-dehydro-TxB_2_ in the lungs. PGD_2_ and 11-dehydro-TxB_2_ stimulate the DP2 receptor on Th2 and ILC2 cells leading to release of type 2 cytokines, IL-4 and IL-13. Both TP activation and IL-4 promote vascular permeability thereby exacerbating fluid transudation, while IL-13 induces hyaluronic acid accumulation and mucus hypersecretion. Ramatroban inhibits the DP2 and TP receptors, thereby promoting pulmonary vasorelaxation and bronchorelaxation and improving capillary barrier function, while attenuating the maladaptive type 2 immune response and mucus secretion, thereby alleviating pulmonary edema and ARDS. Tx, thromboxane; PG, prostaglandin; TP, thromboxane prostanoid receptor; DP2; D-prostanoid receptor 2; Th2; T helper 2; ILC2; innate lymphoid class.

Lungs in COVID-19 patients with acute respiratory distress syndrome (ARDS) produce proinflammatory lipid mediators with predominance of cyclooxygenase metabolites in plasma and bronchoalveolar lavage fluid (BALF), notably thromboxane B_2_ (TxB_2_) >> prostaglandin E_2_ (PGE_2_) > prostaglandin D_2_ (PGD_2_) (Archambault et al., 2021; Regidor et al., 2021). The massive increase in TxA_2_ metabolites in BALF (Archambault et al., 2021) and systemically (Regidor et al., 2021) in hospitalized COVID-19 patients (Hottz et al., 2020; Al-Hakeim et al., 2021; Regidor et al., 2021), suggests a critical role for TxA_2_/TxA_2_ prostanoid receptors (TP) in COVID-19 respiratory distress. We hypothesized that TxA_2_/TP-induced selective contraction of pulmonary veins (Kadowitz et al., 1977; Bowers et al., 1979; Walch et al., 2001) elevates pulmonary capillary pressure and contributes to pulmonary edema and hypoxemia in COVID-19 pneumonia (Figure 1). TP signaling leads to selective constriction of intrapulmonary veins and small airways with 10-fold higher potency and greater reduction in luminal area than intrapulmonary arteries (Larsson et al., 2011). High local concentrations of TxA_2_ can effectively divert pulmonary blood flow, increase microvascular pressure and permeability, and force fluid and plasma proteins into alveoli (Larsson et al., 2011). A selective TP antagonist was previously reported to decrease pulmonary capillary pressure by selectively reducing postcapillary resistance in patients with acute lung injury (Schuster et al., 2001). F2-Isoprostane is also increased in COVID-19 (Kumar et al., 2021) and is known to activate vascular TP receptors. TxA_2_ and isoprostanes stimulate TP-mediated activation of the TGFβ pathway (Craven et al., 1996), and early, untimely TGFβ responses in SARS-CoV-2 infection limit antiviral function of natural killer (NK) cells and promote progression to severe COVID-19 disease (Witkowski et al., 2021).

Theken and FitzGerald proposed early administration of a TxA_2_ receptor antagonist as an antithrombotic agent and a D-prostanoid receptor 2 (DP2, formerly referred to as CRTH2) antagonist to boost interferon lambda (IFN-λ) in the upper respiratory tract, thereby limiting SARS-CoV-2 replication and transmission (Sposito et al., 2021; Theken and Fitzgerald, 2021). Ramatroban, the only dual TxA_2_/TP and PGD_2_/DP2 receptor antagonist available for clinical study, has been proposed as an antithrombotic and immunomodulator agent in COVID-19 (Gupta and Chiang, 2020; Gupta et al., 2020). In their report showing very high levels of TxB_2_ >> PGD_2_ in BALF, Archambault and colleagues also suggested ramatroban to block the deleterious effects of TxA_2_ and PGD_2_ in COVID-19 (Archambault et al., 2021). Ramatroban has an established safety profile, having been prescribed for over 20 years in Japan for treatment of allergic rhinitis (Ishizuka et al., 2004; Uller et al., 2007). We report here a small case series of four consecutive COVID-19 patients with worsening respiratory distress and hypoxemia who were treated with ramatroban. Surprisingly, this led to rapid improvement in both respiratory distress and hypoxemia, thereby avoiding hospitalization and promoting recovery from acute disease.

### 1.1 The First Case of Severe COVID-19 Pneumonia Treated with Ramatroban

S.D., an 87-year-old Indian lady, experienced sudden onset of fever, cough, diarrhea, anorexia, profound weakness, and slight shortness of breath, 10 days after a 2-h flight from New Delhi to Indore, Madhya Pradesh, India. The index patient had received the first dose of COVAXIN, a whole virion inactivated vaccine against SARS-CoV-2, 30 days prior to beginning of symptoms. On examination the patient was fully alert, oriented, and able to make intelligent conversation but lay listlessly in bed unable to ambulate. Patient weighed 42 kg and exhibited severe preexisting muscle wasting and marked kyphosis. Vital signs revealed temperature 102°F, heart rate 100 per minute, blood pressure 90/60 mm of Hg, and respiratory rate 22 per minute. Mucosa were moist, and mild pallor was present. There was no jugular venous distention or pedal edema. Chest examination revealed bilateral coarse rales especially prominent at both lung bases but no wheezes. Abdomen, cardiovascular, and neurological examinations were unremarkable. Patient was not taking any medications.

#### 1.1.1 Past Medical History

Past medical history included hypertension for over 40 years, thyrotoxicosis for over 30 years treated with radioiodine therapy in 1999, severe osteoporosis with kyphosis, bladder suspension surgery in 1999, coronary artery disease leading to acute myocardial infarction and cardiac arrest in 2015 which required coronary angioplasty and stent placement, and chronic kidney disease with estimated glomerular filtration rate of about 20 ml/min (Table 2).

#### 1.1.2 Investigations

Nasopharyngeal and oropharyngeal swabs were positive for SARS-CoV-2 infection by RNA PCR with cycle threshold (Ct range) < 20 cycles. Pulse oximetry revealed oxygen saturation of about 85–88%. Patient was admitted on 9 April 2021 to Medanta Hospital, Indore. CT scan revealed moderate multifocal, patchy ground glass opacities, and consolidation. There was septal thickening in the central and peripheral subpleural aspect of both lung parenchyma. Serial laboratory examinations during the course of the illness are listed in Table 1.

**TABLE 1:** Serial laboratory values of patient S.D.

Analyte	Before admission	During hospital stay	After discharge	Reference value
	8 April 2021	13 April 2021	16 April 2021	
Hemoglobin (g/dl)	11.7	12.1	12.0	13.0–17.0
Platelet count (per mm^3^)	214,000	285,000	402,000	150,000–410,000
WBC count (per mm^3^)	5040	12100	9010	4000–10000
Neutrophils (%)	77	80	86	38–70
Lymphocytes (%)	18	11	07	21–49
NLR (Neutrophil–lymphocyte ratio)	4.3	7.3	12.3	1.1–3.5
Serum CRP (mg/L)	7.86	35.9	15.3	0–5.0
D-Dimer (ng FEU/mL)	600	650	659	<500

#### 1.1.3 Hospital Course

During the hospital stay, the patient was treated with high-flow nasal oxygen, prophylactic low-molecular weight heparin, intravenous remdesivir, antibiotics, and methylprednisolone. Patient continued to have fever, cough, shortness of breath, diarrhea, and profound weakness during the hospital stay. SpO2 on room air ranged between 82 and 86% (Table 2). After a hospital stay of 5 days, the patient was discharged upon her request on 14 April 2021. Discharge medications included oral oseltamivir, doxycycline, vitamin C, aspirin 75 mg once a day, 5 mg prednisolone, vitamin D_3_, and nebulization with budesonide and salbutamol twice daily. Continued supportive management with betadine gargles, steam inhalation, and breathing exercises was advised.

**TABLE 2:** Clinical course of COVID-19 patients with acute respiratory distress treated with ramatroban.

Patient initials, gender, age (years), and comorbidity	Ramatroban (Baynas®) 75 mg tab	Clinical course; blood oxygen saturation by pulse oximetry (SpO_2_)		
		Time “0” ramatroban started	Time to partial relief of dyspnea; and the first SpO2 recorded on room air after initiating ramatroban treatment	Day “5” after taking 10 tablets of ramatroban^b^
S.D.; female, 87 years. Hypertension; Stage 4 CKD; CAD, MI, and cardiac arrest 4 years ago	37.5 mg (½ tab) twice daily	Dyspnea ++ SpO2 : 82%	24–36 h; SpO2 > 90%, 36 h after first dose^a^	No dyspnea SpO2 ≥ 95%
A.K., male, 33 years. Hypertension, psoriasis, and recurrent URTI	75 mg twice daily	Dyspnea +++ SpO2 : 73%	1–2 h; SpO2 90%, 9 h after first dose^b^	No dyspnea SpO2 96%
S.B., female, 22 years	75 mg twice daily	Dyspnea ++ SpO2 : 85%	4–6 h; SpO2 89%, 6–8 h after first dose	No dyspnea SpO2 94%
B.C., male, 70 years. Diabetes mellitus	75 mg twice daily	Dyspnea ++ SpO2 : 80%	2–3 h; SpO2 85%, 2–3 h after first dose	No dyspnea SpO2 96%

a SpO2 on room air was not checked at earlier time points.

b For patients 2, 3, and 4, ramatroban could be administered only for a total of 5 days due to limited supplies.

#### 1.1.4 Postdischarge Course

On April 15, the day after discharge from the hospital, the patient had fever with a temperature of 101°F. Pulse oximetry revealed an oxygen saturation (SpO2) of 82–84% on room air, and patient was continued on oxygen. Patient was profoundly weak and unable to get out of bed without assistance. At this time all drugs including low-dose aspirin were discontinued, and the patient was started on ramatroban (Baynas®, 75 mg tablet) in a dose of one-half tablet (37.5 mg) orally twice daily. The patient was continued on oxygen using a nasal cannula and SpO2 was not checked on room air. After about 36 h, having received three one-half doses of ramatroban, there was noticeable improvement in her general condition, and SpO2 increased to 90% on room air. The dose of ramatroban was increased to 37.5 mg in the morning and 75 mg at bedtime. Patient had complete resolution of cough and diarrhea over the next 3 days and started ambulating independently without assistance. Ramatroban was discontinued after 2 weeks due to nonavailability, and the patient was switched to 75 mg aspirin daily. Patient had recovered almost completely by 22 April 2021 and gradually recovered fully over the next 3–4 weeks to baseline status. On 10 October 2021, 6 months after the acute COVID-19, a high-resolution, noncontrast CT scan demonstrated nonhomogenous ground glass pattern with normal lung volumes and absence of lung fibrosis. Patient continues to be asymptomatic.

### 1.2 Case 2

A.K., a 33-year-old business manager in New Delhi, developed sore throat, cough, loss of smell, altered taste, loss of appetite, high grade fever (104–106°F), profound weakness, and severe body aches around 17 April 2021. A.K. had not received the COVID-19 vaccine. Patient has past medical history of mild hypertension, psoriasis and psoriatic arthropathy treated with homeopathy, nasal polyposis, and recurrent upper respiratory infections every winter for the past several years. Nasopharyngeal and oropharyngeal swabs taken the next day were positive for SARS-CoV-2 infection by RNA PCR with cycle threshold (Ct range) of 21 cycles.

On 18 April 2021, the patient developed progressive shortness of breath and was started on oral favipiravir, hydroxychloroquine, doxycycline, and multivitamins. SpO2 checked in the morning was about 90%, declining to 82–85% by the evening. The shortness of breath worsened around midnight and patient could not catch his breath, was unable to speak, and was very anxious and restless. The SpO2 was 73% (Table 2). Patient could not be transferred to a COVID hospital due to nonavailability of hospital beds.

Desperate attempts to secure an oxygen cylinder failed. Ramatroban was rushed to patient’s home by Uber and first dose of 75 mg was taken at 1:30 a.m. on the morning of April 19th. The “breathing improved in 25–30 min,” and the patient calmed down and fell asleep at 3 a.m. Pulse oximetry remained disconnected while the patient was sleeping so as not to disturb him. Patient woke up at 11 a.m. at which time SpO2 on room air was 88–90%. On April 20th, oral temperature was 101°F and SpO2 was 90–92%. Ramatroban was administered in a dose of 75 mg twice daily for a total of 5 days with continued improvement (Table 2). On the 25th of April, patient noticed that the sputum was streaked with blood and oral acetylcysteine was started. A chest CT on April 27th revealed ground glass opacities involving bilateral lung fields with mild interstitial thickening giving the appearance of crazy-paving pattern. There were scattered areas of bronchopneumonic changes and consolidation involving both lungs. A few small fibrotic bands were noted in both lower lobes. The patient had made a near complete recovery by May 5th and resumed work on May 10th. Patient continued to have altered taste and smell 7 months after the acute illness, but by one year was back to normal.

### 1.3 Case 3

S.B., a 22-year-old, healthy lady in New Delhi developed fever, cough, loss of smell and taste, and body aches due to COVID-19. S.B. had not received COVID vaccination. S.B. was treated with favipiravir, steroids, and multivitamins. Patient experienced progressively worsening shortness of breath and SpO_2_ dropped to 85% on room air. Patient was prescribed Ramatroban 75 mg twice daily. Within 6–8 h after taking the first dose of ramatroban, respiratory distress improved and the SpO2 increased to 89%.

The next day SpO2 increased to 90–91%. There was progressive improvement with complete resolution of respiratory symptoms over the next 5 days. On day 5, the SpO_2_ was 94% on room air (Table 2). Patient has made a complete recovery from acute COVID-19.

### 1.4 Case 4

B.C., a 70-year-old man living in a rural area of Bihar, India, developed high grade fever and cough presumably secondary to SARS-CoV-2 infection. Patient has a history of diabetes mellitus controlled with diet. B.C. was not taking any medications and had only received one dose of COVAXIN vaccine for COVID-19. Patient developed shortness of breath with SpO2 measuring about 80% on room air. Two to three hours after taking 75 mg ramatroban, respiratory distress and cough improved and the SpO2 increased to 85%. After a total of 10 tablets taken over 5 days, dyspnea had resolved and SpO2 increased to 96% on room air (Table 2). Patient has made a complete recovery from acute COVID-19.

### 1.5 Discussion

We present the first reported cases of COVID-19 treated with ramatroban (Baynas®), a dual antagonist of the TxA_2_/TP and PGD_2_/DP2 receptors. All four COVID-19 patients were characterized by respiratory distress that was new in onset or had worsened (Table 2). Despite presenting with severe hypoxemia, gas exchange rapidly improved in all four patients. They were able to avoid hospitalization and recovered without any further need for supplemental oxygen or corticosteroids.

Relief of hypoxemia is a key success factor in COVID-19 treatment. The rapidity of improvement following oral ramatroban is surprising. For COVID-19 patients needing supplemental oxygen outside the intensive care unit in Germany, Daher reported, “The single most outstanding finding of this study is the length of hospitalization and the need of supplemental oxygen: patients were treated for 12 days and needed oxygen therapy for 8 days on average.” Compared to patients being hospitalized for severe influenza, “patients with COVID-19 seem to need a significantly longer hospital stay and are longer on oxygen therapy” (Daher et al., 2021). In India, the PLACID trial evaluated convalescent plasma in patients with moderate COVID-19 and found “median (interquartile range) total days of respiratory support of 9 (6–13) *n* = 227 without and 10 (6–13); *n* = 224 with convalescent plasma.” This trial included younger patients; median age in years was 52 (42–60). (Agarwal et al., 2020). More rapid improvement in hypoxemia and relief of respiratory distress with ramatroban is consistent with acute relaxation of vascular and airway smooth muscles. We hypothesize that blocking TxA_2_/TP inhibits pulmonary venous constriction, lowers pulmonary capillary pressure, and relaxes bronchial smooth muscle. We envision a TP-dependent pressure gradient across the pulmonary microvasculature that forces fluid from the vascular compartment into the alveoli, which together with a TP-dependent increase in microvascular permeability overwhelms lymphatics and floods the small airways, leading to pulmonary edema (Figure 1).

U-46619, a PGH_2_ analog TP agonist, at 1 nM reduced guinea-pig pulmonary venous and airways luminal areas by 50% with little or no change in arterial luminal area (Larsson et al., 2011). Higher concentrations collapsed both pulmonary veins and airways, indicating that subnanomolar concentrations of the more potent TxA_2_ could produce meaningful increases in airway tone and pulmonary venous resistance (Larsson et al., 2011). This is consistent with the measured effect of ifetroban, a selective TP antagonist which reduced pulmonary venous resistance and capillary pressure in patients with acute lung injury (Schuster et al., 2001). Moreover, TP antagonism prevented hypoxemia in a lethal porcine septic shock model (Slotman et al., 1984), attenuated airway mucus hyperproduction induced by cigarette smoke (An et al., 2013), and reduced pulmonary edema in mouse models of acute lung injury (Kobayashi et al., 2016). In the patient cases presented here, we hypothesize that ramatroban enhanced pulmonary blood flow, reduced pulmonary capillary pressures, improved ventilation-perfusion matching, promoted resolution of edema, reduced bronchoconstriction and airway mucus hyperproduction, and improved gas exchange, thereby mitigating SARS-CoV-2 respiratory distress and hypoxemia (Figure 1 and Table 3).

**TABLE 3:** Proposed effect of antagonizing thromboxane A_2_/TP and prostaglandin D_2_/DP2 signaling by ramatroban in patients with COVID-19.

COVID-19	Thromboxane A_2_/TP	Prostaglandin D_2_/DP2
Endogenous agonists for the receptors	Thromboxane A_2_, F2-isoprostane, prostaglandin H_2_	Prostaglandin D_2_, 11-dehydro-thomboxane B_2_
Acute effects of antagonism (minutes-hours)	Hypoxemia ↓, V/Q mismatch ↓, bronchoconstriction ↓, pulmonary edema ↓,	Hyaluronan accumulation ↓,
	⇑	⇑
	pulmonary microvascular permeability ↓, pulmonary capillary pressure ↓, pulmonary venous constriction ↓,	IL-13 ↓,
	NK cell SARS-CoV-2 killing ↑,	antiviral,
	⇑	⇑
	TGFβ ↓	IFN-λ ↑
Subacute short-term effect of antagonism (days–weeks)	Thrombosis ↓	Antiviral activity
		⇑
	Anti-inflammatory (thromboinflammation ↓)	Th1 response ↑, Th2 response ↓
Long-term effect of antagonism (weeks–months)	Brain fog ↓, headache ↓, brain edema ↓,	Depression ↓, activity ↑
	⇑	
	blood–brain barrier ↑,	
	lung fibrosis ↓,	
	⇑	
	TGFβ ↓	

Lung TxA_2_ generation is sufficiently elevated in symptomatic COVID-19 that TP activation may affect other critical organ functions. For example, vascular effects might include vasospasm and thrombosis resulting in renal injury, angina, arrhythmias, myocardial infarction, and/or stroke (Bauer et al., 2014). In the cerebral circulation, TP activation can increase blood–brain barrier permeability (Zhao et al., 2017), which may contribute to headache and brain fog in COVID-19. The potential of TP blockade to affect the lungs and other critical organs during acute illness and during convalescence or long COVID merits focused research.

PGD_2_/DP2 signaling promotes allergic inflammation by stimulating Th2 and innate lymphocyte class 2 (ILC2) cells as in asthma (Figure 1) (Xue et al., 2005; Xue et al., 2014). The maladaptive immune response in COVID-19 is characterized by a shift from Th1 to Th2 with basophilia, eosinophilia, lymphopenia, and an increase in plasma levels of type 2 cytokines produced by Th2 cells, including IL-4 and IL-13 (Lucas et al., 2020; Perlman, 2020; Yang et al., 2020). IL-4 is known to impair the barrier function of endothelial cells, leading to microvascular leakage and edema formation (Figure 1) (Skaria et al., 2016). IL-13 increases hyaluronan accumulation in mouse lungs (Donlan et al., 2021) and mucus overproduction in cultured human bronchial epithelial cells (Tanabe et al., 2008). This is correlated with ARDS, need for mechanical ventilation, acute kidney injury (AKI), and mortality in COVID-19 (Gómez-Escobar et al., 2021). Whether ramatroban inhibits inflammation and hyaluronan accumulation in ARDS remains to be investigated.

The beneficial effects of ramatroban may be additionally attributed to enhanced antiviral activity due to TxA_2_/TP and PGD_2_/DP2 antagonism. First, TxA_2_/TP activation stimulates activation of the TGF-β pathway (Craven et al., 1996), and early, untimely TGF-β responses in SARS-CoV-2 infection limit antiviral function of natural killer (NK) cells (Witkowski et al., 2021). Second, TxA_2_/TP activation may directly modulate natural cytotoxic effector cell function (Rola-Pleszczynski et al., 1985). Third, PGD_2_/DP2 signaling may suppress innate mucosal antiviral responses by inhibiting expression of interferon (IFN)-λ, the first line of defense against viruses at mucosal surfaces. Notably, IFN-λ is markedly suppressed in the upper respiratory tract in COVID-19 patients (Broggi et al., 2020). Increased expression of phospholipase A_2_ group IID and PGD_2_ in the elderly may further suppress IFN-λ expression (Werder et al., 2018), thereby impairing antiviral responses and contributing to increased morbidity and mortality observed consistently in the elderly (Sposito et al., 2021). Expression of nasal and pharyngeal PGD_2_ and DP2 in SARS-CoV-2 infection remains to be investigated even though there is significant elevation of PGD_2_ in bronchoalveolar lavage fluid and human lung epithelial cells (Archambault et al., 2021; Ricke-Hoch et al., 2021) and expression of PGD_2_ synthase and DP2 in COVID-19 kidneys (Diao et al., 2021).

Interestingly, 11-dehydro-TxB_2_ (11dhTxB_2_), a major stable metabolite of thromboxane A_2_, serves as a full agonist of DP2 receptors (Böhm et al., 2004). Urinary 11dhTxB_2_ levels are markedly increased in COVID-19 and correlate with length of hospitalization, mechanical ventilation, and mortality (Tantry et al., 2021). In rabbits infused with TxB_2_, 11dhTxB_2_ was a major metabolite, and enzymatic conversion of TxB_2_ to 11dhTxB_2_ was not detected in blood cells or plasma (Westlund et al., 1986). The dehydrogenase catalyzing formation of 11dhTxB_2_ was tissue bound with the highest activity in lung (Westlund et al., 1986). The above suggests that elevated lung TxA_2_ is converted to 11dhTxB_2_, which may exert local or systemic effects *via* DP2. In a neonatal mouse model of severe respiratory syncytial virus-induced bronchiolitis, treatment with a DP2 antagonist decreased viral load and improved morbidity associated with upregulating interferon (IFN)-λ expression (Werder et al., 2018; Theken and Fitzgerald, 2021). Whether ramatroban enhances NK cell and IFN-λ responses and reduces SARS-CoV-2 viral load remains to be investigated.

Currently, there is no treatment for long-haul COVID symptoms following recovery from acute illness. Long-haul COVID is often characterized by neuropsychiatric manifestations including “brain fog,” anxiety or depression, fatigue and problems with mobility, dyspnea, in part due to lung fibrosis and lung diffusion impairment, and microvascular thrombosis persisting for >4 months in about 25% of patients (Huang et al., 2021; Townsend et al., 2021). Despite persistence of ground glass opacities 6 months later in patient 1, lung fibrosis was not detected. This is consistent with the antifibrotic effect of ramatroban in an animal model of silicosis that is associated with markedly increased pulmonary thromboxane A_2_ and PGD_2_ (Pang et al., 2021). Moreover, in well-established animal models of depression, elevation in PGD_2_ mediates depression-like behavior, while ramatroban restores object exploration and social interaction (Onaka et al., 2015). The above suggests that ramatroban may help prevent and/or treat certain long-haul COVID symptoms (Table 3).

This report has several limitations. Only 4 patients could be treated with ramatroban, and the duration of treatment was brief due to very limited availability of the drug in India. Only the first patient had laboratory studies performed. Patients 2, 3, and 4 were not examined by a physician and the clinical course was reported by patients or their relatives.

During the ongoing pandemic, there is an unmet need for a drug that can provide rapid relief of respiratory symptoms and hypoxemia, halt progression of disease, and minimize hospitalization, which is associated with poor outcomes for the patient and added burden on the healthcare system. Ramatroban (Baynas®, Bayer Yakuhin, Ltd., Japan) has been safely used for the treatment of allergic rhinitis in Japan since 2000 (Ishizuka et al., 2004). The usual adult oral dose of 75 mg twice daily achieves an average plasma concentration of about 0.1 mg/L or 240 nM which is sufficient to inhibit pulmonary venous constriction, platelet activation, and release of type 2 cytokines (Table 3).

Bleeding has emerged as a significant complication in hospitalized COVID-19 patients. The overall and major bleeding rates were 4.8% (95% CI, 2.9–7.3) and 2.3% (95% CI, 1.0–4.2), respectively, among 144 patients with moderate to severe COVID-19 (Al-Samkari et al., 2020). Major bleeding occurred in 3.8% of the patients assigned to therapeutic-dose anticoagulation with heparin and in 2.3% of those assigned to usual-care pharmacologic thromboprophylaxis (The REMAP-CAP et al., 2021). In the 4-patient case series reported here, only one patient (Case 2, A.K.) experienced any evidence of bleeding. He reported blood-tinged sputum >24 h after the last dose of ramatroban, but that was not clinically significant. Because ramatroban’s plasma half-life is about 2 h, platelet-dependent hemostasis is unlikely to be continuously impaired with 75-mg doses given about 12 h apart (Ishizuka et al., 2004; Nippon Shinyaku Co. Ltd, 2009).

The rapid and salutary responses to ramatroban reported here, and its diverse actions targeting the major pathobiologic mechanisms underlying COVID-19 (Table 1 and Figure 1), coupled with its oral bioavailability and an excellent safety profile make ramatroban an attractive therapeutic agent to test in randomized controlled clinical trials.

## Data Availability

The original contributions presented in the study are included in the article/Supplementary Material, and further inquiries can be directed to the corresponding authors.

## Abbreviations

Tx, thromboxane; 11dhTxB2, 11-dehydro-thromboxane B2; TP, thromboxane prostanoid receptor; PG, prostaglandin; COX, cyclooxygenase; DP2, D prostanoid receptor 2; URTI, upper respiratory tract infection; BALF, bronchoalveolar lavage fluid; SARS-CoV-2, severe acute respiratory syndrome coronavirus 2; COVID-19, coronavirus disease 2019; RSV, respiratory syncytial virus; IL, interleukin; MDSC, monocyte–macrophage-derived suppressor cell; IFN, interferon; ARDS, acute respiratory distress syndrome; SpO2, blood oxygen saturation by pulse oximetry; TGF-β, transforming growth factor beta; NK cells, natural killer cells; NETs, neutrophil extracellular traps.
